# Top-Down Predictions of Familiarity and Congruency in Audio-Visual Speech Perception at Neural Level

**DOI:** 10.3389/fnhum.2019.00243

**Published:** 2019-07-12

**Authors:** Orsolya B. Kolozsvári, Weiyong Xu, Paavo H. T. Leppänen, Jarmo A. Hämäläinen

**Affiliations:** ^1^Department of Psychology, University of Jyväskylä, Jyväskylä, Finland; ^2^Jyväskylä Centre for Interdisciplinary Brain Research (CIBR), University of Jyväskylä, Jyväskylä, Finland

**Keywords:** speech perception, magnetoencephalography, audio-visual stimuli, audio-visual integration, familiarity

## Abstract

During speech perception, listeners rely on multimodal input and make use of both auditory and visual information. When presented with speech, for example syllables, the differences in brain responses to distinct stimuli are not, however, caused merely by the acoustic or visual features of the stimuli. The congruency of the auditory and visual information and the familiarity of a syllable, that is, whether it appears in the listener’s native language or not, also modulates brain responses. We investigated how the congruency and familiarity of the presented stimuli affect brain responses to audio-visual (AV) speech in 12 adult Finnish native speakers and 12 adult Chinese native speakers. They watched videos of a Chinese speaker pronouncing syllables (/pa/, /pha/, /ta/, /tha/, /fa/) during a magnetoencephalography (MEG) measurement where only /pa/ and /ta/ were part of Finnish phonology while all the stimuli were part of Chinese phonology. The stimuli were presented in audio-visual (congruent or incongruent), audio only, or visual only conditions. The brain responses were examined in five time-windows: 75–125, 150–200, 200–300, 300–400, and 400–600 ms. We found significant differences for the congruency comparison in the fourth time-window (300–400 ms) in both sensor and source level analysis. Larger responses were observed for the incongruent stimuli than for the congruent stimuli. For the familiarity comparisons no significant differences were found. The results are in line with earlier studies reporting on the modulation of brain responses for audio-visual congruency around 250–500 ms. This suggests a much stronger process for the general detection of a mismatch between predictions based on lip movements and the auditory signal than for the top-down modulation of brain responses based on phonological information.

## Introduction

In most cases speech perception relies on the seamless interaction and integration of auditory and visual information. Listeners need to efficiently process a rapid and complex stream of multisensory information, making use of both visual and auditory cues. We wanted to examine how lifelong exposure to audio-visual speech affects the brain mechanisms of cross-modal integration and mismatch. Auditory and visual cues can be presented either congruently or incongruently and this match or mismatch of features could be used to study the audio-visual processing of speech. Using magnetoencephalography (MEG), we studied how the effects of congruency and familiarity (i.e., whether the speech stimuli are part of the listener’s phonology or not) of the auditory and visual features are reflected in brain activity.

Audio-visual speech has been shown to activate (in sequence) the sensory areas around 100 ms from stimulation onset in the auditory and visual cortices (Sams et al., 1991; Möttönen et al., 2004; Salmelin, 2007), then the superior temporal sulcus around 150 ms (Nishitani and Hari, 2002), which has been shown to play an important role in the perception and interpretation of movements (both facial and body) of the speaker (Puce et al., 1998; Iacoboni et al., 2001). The inferior parietal cortex has been shown to be activated at around 200 ms, which is suggested to be related to the connection of the STS to the inferior frontal lobe (Broca’s area) (Nishitani and Hari, 2002) with stronger activations in the left hemisphere than in the right (Capek et al., 2004; Campbell, 2008). This is followed by activation in the frontal areas close to Broca’s area around 250 ms (Nishitani and Hari, 2002).

It has been suggested (Campbell, 2008) that seeing speech can affect what is perceived in either a complementary or correlated way. In the complementary mode, vision offers further information about some aspects of speech, which are harder to detect only auditorily and which may depend on the clear visibility of the speaker’s lower face. In the correlated mode, on the other hand, successful speech processing depends on the speech stream’s temporal-spectral signature showing similar dynamic patterning across both the audible and visible channels.

Audio-visual mismatch is often examined from the point of view of congruency (Jones and Callan, 2003; Hein et al., 2007), where congruent and incongruent audio-visual pairs are contrasted. The assumption is that congruency should only have an effect on perception when the inputs of unimodal sources have been integrated (van Atteveldt et al., 2007). In terms of brain responses, the STS has been shown to be a critical brain area for multisensory integration and congruency of auditory and visual information in the case of both speech and non-speech stimuli. For example, Beauchamp et al. (2010) used TMS to disrupt brain activity in STS, while participants viewed audio-visual stimuli that have been shown to cause the McGurk effect (where incongruent auditory and visual speech cues presented together produce an illusory percept; McGurk and Macdonald, 1976). When TMS was applied to the left STS during the perception of McGurk pairs, the frequency of the McGurk percept was greatly reduced. This reduction, in the likelihood of the McGurk effect, demonstrates that the STS is an important cortical locus for the McGurk effect and plays an important part in auditory-visual integration in speech.

Furthermore, a broad network of brain regions in addition to the STS have been found in fMRI studies to show differences between brain responses to incongruent and congruent audio-visual speech, including the precentral gyrus (Jones and Callan, 2003), the inferior parietal lobule (Jones and Callan, 2003), the supramarginal gyrus (Jones and Callan, 2003), the superior frontal gyrus (Miller and D’Esposito, 2005), Heschl’s gyrus (Miller and D’Esposito, 2005) and the middle temporal gyrus (Callan et al., 2004).

Previous studies examining audio-visual speech have found relatively early event-related brain potential (ERP) effects around N1 and P2 responses (Stekelenburg and Vroomen, 2007; Baart et al., 2014). In this case the visual information leads the auditory information, that is, lip movements can precede actual phonation for up to several hundreds of milliseconds (Stekelenburg and Vroomen, 2007). This visual information allows the observer to make predictions about several aspects of the auditory signal (e.g., content, timing). Studies have shown that the auditory-evoked N1 and P2 components of ERPs, at latencies of 100–150 and 200–250 ms, respectively, are attenuated and speeded up when the auditory signal is accompanied by visual speech (Klucharev et al., 2003; Besle et al., 2004; van Wassenhove et al., 2005; Stekelenburg and Vroomen, 2007). This suggests early predictive effects of the visual information on the auditory stimulation. Furthermore, no attenuation in N1 was found when no visual anticipatory information about sound onset is present, indicating that the temporal information present in the visual stimulus, rather than the content of the sound, is key in audio-visual interaction (Stekelenburg and Vroomen, 2007; Vroomen and Stekelenburg, 2010).

However, the N1 and P2 responses seem to be sensitive to the stimulus material. This was shown by Baart et al. (2014), who investigated speech-specific audio-visual integration, where they used speech stimuli and sinewave speech, and found that N1 suppression occurs regardless of the type of stimuli, but P2 amplitude was only suppressed in relation to speech stimuli. They found congruency effects for responses to speech stimuli from around 200 ms after audio-visual incongruency became apparent, with ERPs being more negative for congruent stimuli than for incongruent stimuli. These early suppression effects were found when comparing the brain responses between the unimodal and the multimodal stimuli.

In addition, audio-visual speech congruency effects have also been reported in later time-windows. Arnal et al. (2009) investigated how the visual signal of an audio-visual stimulus affects auditory speech processing. In their experiment they recorded early visual and auditory responses to matching (congruent) and non-matching (incongruent) audio-visual syllables using MEG and found no effect of audio-visual incongruence in the early time-window (M100). They detected the earliest mismatch effect 120 ms after voice onset, followed by three more maxima at 250, 370, and 460 ms. Their findings indicated a multistep comparison between the top-down visual prediction and the bottom-up auditory signal.

Another aspect affecting audio-visual speech is the long-term memory representations of speech, that is, the familiarity of the speech itself. It has been documented that speech perception is altered by an individual’s language experience. Iverson et al. (2003) found that listeners of different languages respond to distinct acoustic aspects of the same speech stimulus. They compared Japanese, German, and English speakers’ responses to contrasts of /ra/ and /la/, where they had to rate whether the stimulus presented was a good exemplar of their own native-language phoneme. They found that American listeners attend to the third formant, which reliably distinguishes /r/ from /l/, while Japanese listeners attend more strongly to the second formant, which is critical for distinguishing Japanese phonemes, but is not at all helpful in distinguishing /r/ from /l/.

This and other studies suggest that the effects of language experience on speech perception are due to neural coding of the acoustic components that are critical to native-language processing (e.g., Kuhl, 2000, 2004). Such effects of language exposure are reflected in brain responses around 150–200 ms, for example in the modulation of the strength of the mismatch negativity (MMN), which is thought to tap into language-specific perceptual sensitivity (Näätänen et al., 1997, 2007; Winkler et al., 1999; Zhang et al., 2005, 2009). Language-specific phonetic-phonological analysis has been shown to start 100–200 ms following stimulus onset (Vihla et al., 2000; Näätänen et al., 2007). MMN or mismatch field (MMF) in EEG and MEG studies, respectively, have indicated access to phonological categories (Vihla et al., 2000; Näätänen et al., 2007) and the distinct processing of native and non-native phonetic contrasts (Näätänen et al., 1997, 2007) in this time-window.

By comparing two groups with different native languages (Finnish and Chinese), we aimed to see how long-term audio-visual representations affect speech perception by examining the congruency effects. Additionally, we aimed to distinguish the effects of familiarity, which is a learned aspect of speech, from congruency, which should be an inherent aspect of the audio-visual stimuli related to the general correspondence between mouth movements and speech signal.

To this end, we compared brain responses measured with MEG to unfamiliar and familiar (called aspirated and unaspirated, respectively, see section “Materials and Methods” below) and also congruent and incongruent audio-visual speech stimuli. We expected to find significant differences in responses to congruent and incongruent stimuli for both Chinese and Finnish participants with larger responses to incongruent stimuli starting from 150 ms or later based on the previous literature (e.g., Arnal et al., 2009). However, in the case of the Finnish participants, we expected differences between the familiar and unfamiliar stimuli specifically starting in the same time-window as the congruency effect (150 ms onward), with the unfamiliar stimuli producing a larger response than the familiar stimuli if long-term phonological representations facilitate the processing of audio-visual speech.

## Materials and Methods

### Participants

Participants were adult Finnish native speakers and adult Chinese native speakers studying in Jyväskylä, Finland. None of the participants had neurological or learning problems, hearing difficulties, using medication affecting the central nervous system, head injuries, ADHD or language-specific disorders. They all had normal or corrected-to-normal sight. The Finnish participants had no exposure to the Chinese language. In total, 19 Finnish native speakers and 18 Chinese native speakers participated in the study. Of these, 13 were excluded from the analysis due to excessive head movement (two participants), poor vision after correction (two participants), technical problems during recording (three participants), strong noise interference (two participants), or otherwise bad signal quality (four participants). Data included in the analysis were from 12 Finnish participants and 12 Chinese participants (see Table 1 for characteristics of participants included).

**Table 1:** Participant characteristics.

Native language	Finnish	Chinese
Mean age (*SD*)	23.92 (1.98)	24.75 (3.39)
Gender ratio (male:female)	6:6	3:9
Handedness ratio (right:left)	12:0	12:0


*Mean age, gender ratio and handedness are for those included in the analysis.*

Ethical approval for the study was provided by the Ethical Committee of the University of Jyväskylä. Participants gave their written informed consent to participate in the study. All participants received movie tickets as compensation for participating in the study.

### Stimuli

**FIGURE 1:**
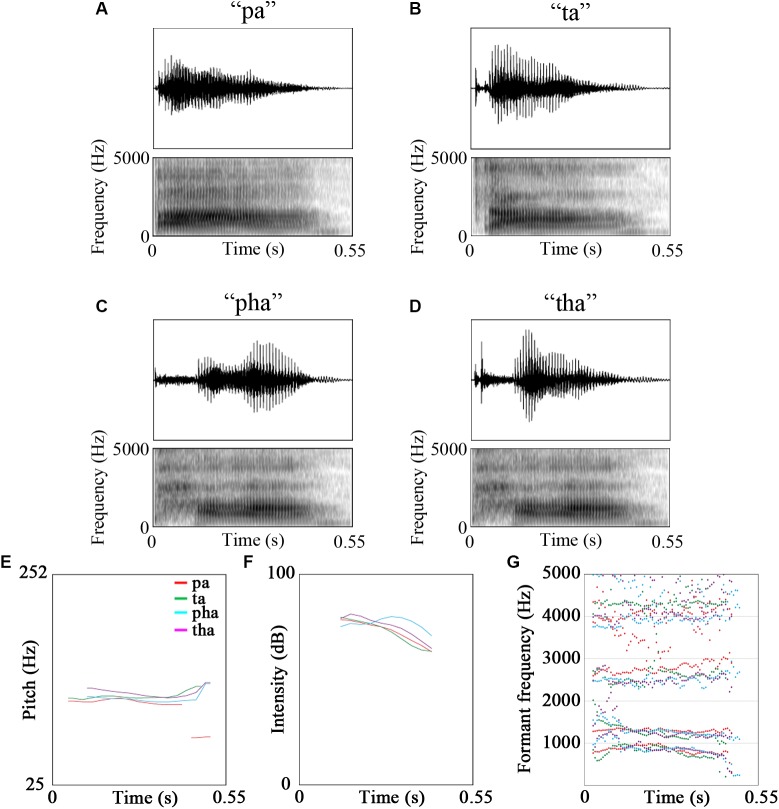
Oscillograms, spectrograms and the acoustic features of the stimuli **(A)** pa, **(B)** ta, **(C)** pha, **(D)** tha, **(E)** pitch, **(F)** intensity, **(G)** formant frequencies (red - /pa/, green - /ta/, cyan - /pha/, purple - /tha/).

The stimuli were video recordings of the syllables /pa/, /pha/, /ta/, /tha/ and /fa/. Of these five syllables, /fa/ was used for a cover task to maintain participants’ attention on the stimuli [see Figure 1 for oscillograms, spectrograms and acoustic features of the stimuli. Figures were created using Praat (Boersma and Weenink, 2018), see Table 2 for description of the stimuli]. The videos were recorded using a Canon Legria HF200 HD video camera and were edited in Adobe Premier Pro CS5.5 to be 1800 ms long. The videos were recordings of a male native Mandarin Chinese speaker.

**Table 2:** Stimuli description.

Modality	Target	Familiar / Unaspirated		Unfamiliar / Aspirated	
Audio	fa A	pa A	ta A	pha A	tha A
Visual	fa V	pa V	ta V	pha V	tha V
AV congruent	fa V / fa A	pa V / pa A	ta V/ ta A	pha V /pha A	tha V / tha A
AV incongruent	–	pa V / tha A	ta V / pha A	pha V / ta A	tha V / pa A


For the Finnish participants, /pa/ and /ta/ were considered familiar stimuli because they are part of their native phonology. For the Chinese participants all four syllables were familiar. The recordings could be audio only, in which the participant was presented with the audio track and the still image of the speaker; visual only, in which the video was presented without any sound; and audio-visual, where both audio track and video were presented at the same time. The audio-visual condition could be congruent, where what they saw was what they heard, or incongruent, where the audio did not match the video.

### Procedure

Participants sat in a magnetically shielded, sound-attenuated room. They sat under the MEG helmet in a 68° sitting position.

Stimuli were presented using Presentation software (version 18.1; Neurobehavioral Systems, Inc., Albany, CA, United States) running on a Microsoft Windows computer using a Sound Blaster Audigy RX sound card and NVIDIA Quadro K5200 video card.

The stimuli were presented on a projector screen. Stimuli were projected from outside of the measurement room onto a mirror then reflected onto the projector screen using a Barco FL35 projector. The participants were sitting 1 m from the projection screen.

The participants were asked to watch short videos of a speaker uttering syllables and to attend to all stimuli presented. The videos were cropped to the mouth area of the speaker (from just above the nose to the clavicles). The fixation cross before the onset of the video clip was centered on where the lips of the speaker were in the videos. Videos were presented on a black background, in the center of the screen. The lights were dimmed. Sounds were presented through insert earphones (Lo-Fi auditory stimulation system, Elekta MEGIN Triux) at ∼70 dB sound pressure level.

The participants were presented with a blank screen for 500 ms, then a fixation cross for 550 ms, followed by a still image of the speaker for 500 ms and finally the stimuli, which was 1800 ms long.

**FIGURE 2:**
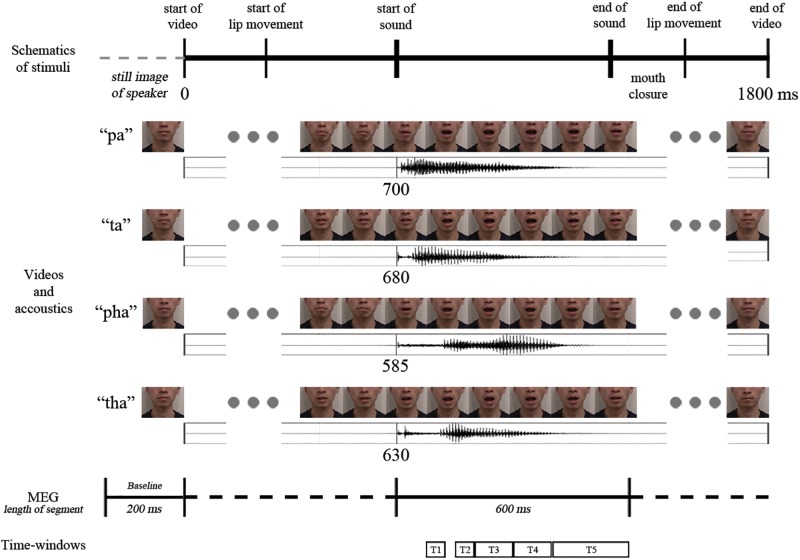
Schematic representation of the temporal structure of the four congruent audio-visual stimuli used (above: video frames, below: oscillogram) and the analysis intervals (marked with T1–T5).

The participants received eight practice trials. In the actual experiment 220 stimuli (20 targets for the cover task, and 50 audio-visual congruent, 50 audio-visual incongruent, 50 audio and 50 visual stimuli; /pa/ and /ta/ repeated 12 times each, /pha/ and /tha/ repeated 13 times each) were presented in pseudo-random order with no immediate repetitions of the same stimuli. Stimuli were presented in two blocks, with a short break (duration determined by the participant) in between the blocks (see Figure 2 for a schematic representation of the video sequence and timings).

As a cover task the participants were asked to press a button to indicate if they saw and/or heard the target syllable /fa/.

### Magnetoencephalography Recording and Preprocessing

The MEG data were recorded by a whole-head 306 channel Elekta Neuromag TRIUX MEG device in Jyväskylä, Finland, including 102 magnetometers and 204 orthogonal planar gradiometers. EOG was measured from two diagonally placed electrodes, slightly above the right eye and slightly below the left eye, with the ground electrode on the right clavicle. Five head position indicator (HPI) coils were attached to the scalp, three on the forehead and one behind each ear, and were used to monitor the location of the head in relation to the sensors during the recording by sending 293, 307, 314, 321, and 328 Hz sinusoidal currents into the five coils, respectively. The Polhemus Isotrak digital tracker system (Polhemus, Colchester, VT, United States) was used to determine the position of the HPI coils in relation to three anatomical landmarks (the nasion, left and right preauricular points). For co-registration purposes an additional set of scalp points (>100) were also digitized, distributed randomly over the skull.

Magnetoencephalography data were collected with a sampling rate of 1000 Hz and an online filter of 0.1–330 Hz. All data were preprocessed using the temporal extension of the signal space separation (tSSS) method with buffers of 30 s (Taulu and Kajola, 2005; Taulu et al., 2005) in Maxfilter 3.0^TM^ (Elekta AB) to remove external interference and correct for head movements. Bad channels were identified by visual inspection and marked for exclusion and reconstructed by the MaxFilter program. Head position was estimated in 200 ms time-windows and 10 ms steps for movement compensation.

Data were preprocessed using MNE Python (0.16.2) (Gramfort et al., 2013). Independent component analysis (ICA) using the fastICA algorithm (Hyvärinen and Oja, 2000) was applied to remove eye blinks, horizontal eye movements and cardiac artifacts. Data were low-pass filtered at 35 Hz using a zero-phase FIR filter with a bandwidth of 8.8 Hz. Then the continuous MEG recording was epoched into 200 ms before to 1800 ms after the onset of the video stimuli in the audio-visual condition. The epoched data were baselined using the 200 ms preceding the onset of stimuli. The epochs were shortened and realigned to 200 ms before and 1000 ms after the start of sound in the audio-visual condition. Data were then manually checked to remove any head movement–related artifacts and electronic jump artifacts. MEG epochs exceeding 2 pT/cm for gradiometer or 4 pT for magnetometer peak-to-peak amplitudes were excluded from further analysis. After artifact rejection, an average of 96.50% of trials were used for analysis. Event-related fields were obtained by averaging trials for different conditions separately. The data were then resampled to 250 Hz to shorten the computation time in the statistical analysis.

Statistical analysis of sensor-level data was done in FieldTrip toolbox (downloaded 20 October 2016; Oostenveld et al., 2011) for MATLAB R2016b (The MathWorks Inc., Natick, MA, 2000) while source-level analyses were run in MNE Python.

### Time-Windows

Based on previous literature, five time-windows were investigated: 75–125, 150–200, 200–300, 300–400, and 400–600 ms (where 0 ms is the start of the sound in the section “Stimuli” as described above). The first time-window encompasses the basic auditory N1 m response (Poeppel et al., 1996; Parviainen et al., 2005; Salmelin, 2007), where the brain extracts speech sounds and their sequences from the incoming auditory signal and the responses are expected to be in the auditory cortices. The second time-window has been shown to be involved in further phonemic processing of the stimulus (Näätänen et al., 1997, 2007; Salmelin, 2007) with responses localized to the temporal cortex. The third time-window has been shown to be responsive to lexical-semantic manipulations (Helenius et al., 2002; Kujala et al., 2004) as well as to audio-visual manipulations (e.g., Raij et al., 2000; Arnal et al., 2009, around 250 ms), as have the fourth (Arnal et al., 2009, around 370 ms; Baart et al., 2014, 300–500 ms after onset of AV congruency) and the fifth time-windows (Arnal et al., 2009, around 460 ms).

### Sensor-Level Analysis

Averaged planar gradiometer data were transformed into combined planar gradients using the vector sum of the two orthogonal sensors at each position implemented in the Fieldtrip toolbox (Oostenveld et al., 2011), which were then used for sensor-level analysis. Gradiometers were chosen because they are less sensitive to noise sources originating far from the sensors than magnetometers are.

Permutation tests with spatial and temporal clustering based on *t*-test statistics were carried out for planar gradients of individual averaged ERFs (Maris and Oostenveld, 2007). The five time-windows defined (see above) were investigated separately, with a cluster α level of 0.05 and the number of permutations 3000.

### Source-Level Analysis

Source analysis was carried out with a minimum-norm estimate on the event-related fields of the magnetometers and gradiometers (Hämäläinen and Ilmoniemi, 1994). The noise covariance matrix was calculated from the baseline period of 200 ms preceding the start of the video (i.e., the participants were viewing the still image of the speaker).

Individual magnetic resonance images (MRI) were not available from the participants and therefore Freesurfer (RRID:SCR_001847) average brain (FSAverage) was used as a template for the source analysis (see below). Three-parameter scaling was used to co-register FSAverage with individual digitized head points. The average co-registration error was 3.54 mm (*SD*=0.27). A single layer BEM (Boundary Element Method) solution was used for the forward modeling.

Depth-weighted L2-minimum-norm estimate (wMNE) (Hämäläinen and Ilmoniemi, 1994; Lin et al., 2006) was calculated for 4098 current dipoles with free orientation distributed on the cortical surface in each hemisphere. Dynamic statistical parametric mapping (dSPM) (Dale et al., 2000) was used to noise-normalize the inverse solution for further statistical analysis. Cluster-based permutation statistics in MNE Python were run on the dSPM source waveforms.

### Statistical Analyses

Accuracy and reaction times in the cover task were examined using Target type (Audio only, Visual only, Audio-Visual) by Native language (Finnish, Chinese) ANOVAs.

Congruency and familiarity effects were examined using the interaction of Stimulus by Native language by comparing difference waves between the groups. If no significant results were obtained, Stimulus main effects were investigated between the stimuli. For comparisons investigating congruency, we compared responses to the congruent and incongruent audio-visual stimuli. For comparisons investigating familiarity, we compared responses to the congruent unaspirated audio-visual (/pa/ and /ta/ syllables) and the congruent aspirated audio-visual (/pha/ and /tha/ syllables) stimuli.

## Results

### Behavioral Performance

Participants’ accuracy scores were close to 100% (Finnish: 97.88%; Chinese: 98.35%) (Table 3), indicating that they were indeed paying attention to the stimuli. Accuracy (percentage of correct responses) were averaged for each participant, and a 3 (Target type: Audio only, Visual only, Audio-Visual) × 2 (Native language: Finnish, Chinese) repeated measures ANOVA resulted in no significant interaction or main effects.

**Table 3:** Accuracy scores for the Finnish and Chinese participants in detecting the target syllable /fa/.

				Accuracy (% of correct response to the target stimulus)
	AV stimuli (%)	A stimuli (%)	V stimuli (%)	All stimuli (%)
Finnish (*n* = 12)	100	97.22	96.43	97.88
Chinese (*n* = 12)	98.81	98.61	97.62	98.35
Total (*n* = 24)	99.40	97.92	97.02	98.12


Reaction times were on average 1189.72 ms (*SD*: 125.86) (Table 4). Reaction times were averaged for each participant, and a 3 (Target type: Audio only, Visual only, Audio-Visual) × 2 (Native language: Finnish, Chinese) repeated measures mixed ANOVA resulted in a significant Target type main effect [*F*(1.954,42.985) = 6.338, *p* = 0.004, partial η2 = 0.224]. *Post hoc t* tests revealed that there was a significant difference between response time to visual only and audio only targets [*t*(23) = 2.943, *p* = 0.007], and audio-visual and audio only targets [*t*(23) = 3.518, *p* = 0.002] with audio only targets having longer reaction times than the other targets.

**Table 4:** Reaction times for the Finnish and Chinese participants in detecting the syllable /fa/.

				Reaction times in ms (*SD*)
	AV stimuli	A stimuli	V stimuli	All stimuli
Finnish (*n* = 12)	1170.56 (94.06)	1230.43 (94.51)	1187.56 (141.20)	1193.69 (103.84)
Chinese (*n* = 12)	1152.81 (151.20)	1201.29 (83.87)	1142.48 (160.23)	1163.16 (127.68)
Total (*n* = 24)	1161.69 (123.48)	1215.86 (88.64)	1165.02 (149.48)	1178.42 (114.88)


### MEG

Our focus was on the native language interactions and we first examine, and report results with significant native language effects. In the absence of interactions, we report the main effects of congruency and familiarity.

Grand average plots of responses at sensor and source level for the congruency comparison and the familiarity comparison can be seen in Supplementary Figures S1, S2, respectively.

### Sensor-Level Analysis

#### Congruency Effects

No significant effects were found in the first, second, third or fifth time-windows.

**FIGURE 3:**
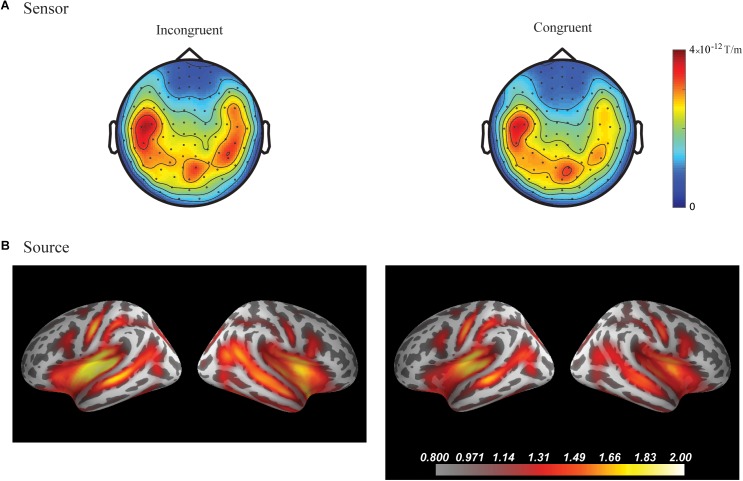
Grand average plots at sensor and source level for incongruent and congruent audio-visual stimuli in the fourth time-window (300–400 ms) for the combined group (Chinese and Finnish speakers, *N* = 24). **(A)** Magnetic field topography of grand average evoked responses from combined planar gradients in the fourth time-window. **(B)** Dynamic statistical parametric maps (dSPM) of source activation of the grand average evoked responses in the fourth time-window.

**FIGURE 4:**
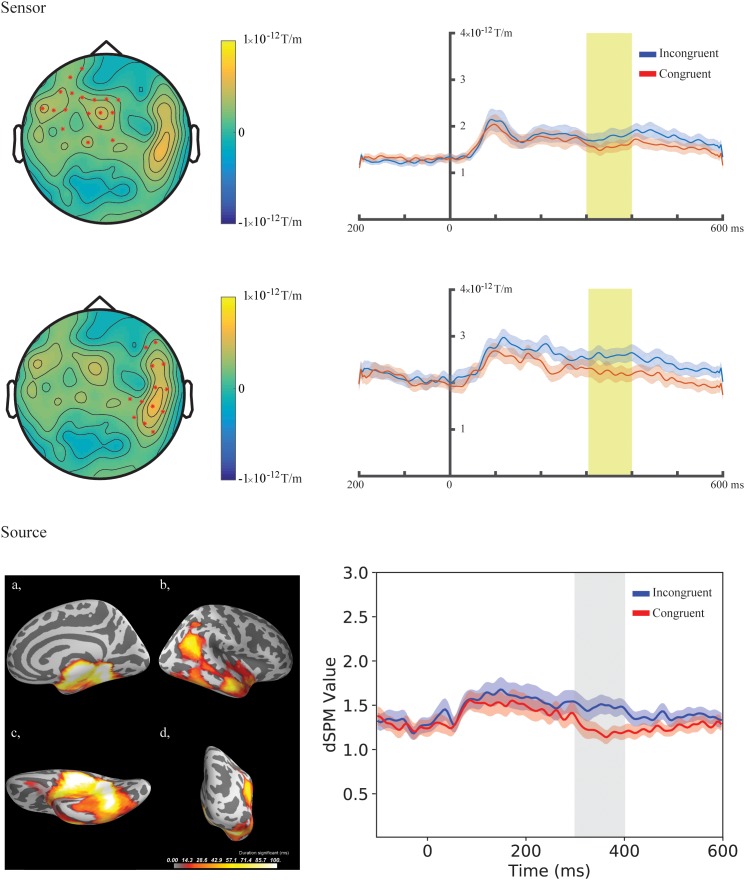
Sensor- and source-level clusters based on the permutation statistics of the congruency effects for the combined group (Chinese and Finnish speakers, *N* = 24). **Left:** Clusters are represented by red dots in the sensor space and yellow and red colouring on the cortical surfaces for the source space. The brightness of the cluster was scaled by the temporal duration of the cluster in the source space. Lower left corner shows the cluster-based permutation statistics results for the incongruent vs congruent comparison in the source space: (a) Medial view, (b) Lateral view, (c) Ventral view, (d) Caudal view. **Right:** average evoked responses from the channels forming the cluster for the sensor space results and the source waveform (dSPM value) extracted from the clusters for the source space results. The red and blue shaded area represents the standard error of the mean and the yellow and gray shaded area indicates the time-window of the cluster.

In the fourth time-window, two clusters were found to be significant for the Congruency main effect (responses to the incongruent stimuli compared to responses to the congruent stimuli) after the cluster permutation tests. One cluster (*p* = 0.036654) was found in the left frontal areas and another cluster (*p* = 0.046651) was found in the right temporal areas. See Figure 3 for the topographic maps of brain responses in this time-window. See Figure 4 for the topographic maps of the clusters and the average evoked responses from the channels forming the clusters.

#### Familiarity Comparison (Audio-Visual)

No significant statistical effects were found in the five time-windows examined using the cluster permutation tests.

### Source-Level Analysis

#### Congruency Effects

No significant differences were found in the first, second, third and fifth time-windows.

In the fourth time-window, one cluster was found to be significant (*p* = 0.039) after the cluster permutation tests for the Congruency main effect (responses to the incongruent stimuli compared to responses to the congruent stimuli). The cluster encompassed the right temporal-parietal and medial areas. See Figure 3 for dynamic statistical parametric maps (dSPM) source activation in this time-window. See Figure 4 for the source waveform (dSPM value) extracted from the significant cluster.

#### Familiarity Comparison (Audio-Visual)

No significant statistical effects were found in the five time-windows examined using the cluster permutation tests.

All non-significant results of the permutation tests in the five time-windows, with lowest *p*-values, are reported in the Supplementary Material 3.

## Discussion

We investigated how the congruency and familiarity of a stimulus could affect audio-visual speech perception in two groups of adults, native speakers of Chinese and those of Finnish. The Chinese participants had long-term exposure to all of the stimuli because they belonged to their native language, but some of the speech sounds were not part of Finnish phonology, thus making them unfamiliar for the Finnish participants. We found significant differences in the congruency comparisons across these groups. A significant congruency main effect was found in the frontal and temporal regions at the sensor level and in the right temporal-parietal regions at the source level 300–400 ms following the onset of sound, but no significant effects were found for familiarity comparisons. Matching and mismatching audio-visual speech thus produces robust and replicable processing differences in the brain, which is consistent with findings in earlier studies. Direct comparison of responses to stimuli familiar (unaspirated) and unfamiliar (aspirated) to the Finnish participants do not show evidence for strong cross-modal top-down predictions that would modulate obligatory sensory brain responses.

We found a significant difference between the responses to the congruent and incongruent stimuli for Chinese and Finnish participants in the 300–400 ms time-window bilaterally at the sensor level at the left frontal and right temporal areas as well as in the right hemisphere at the source level in the temporal-parietal areas, indicating that both groups detected the incongruency. The time-window is in line with similar earlier studies using native language stimuli where the incongruence effects were found around 300–500 ms (Arnal et al., 2009; Baart et al., 2014). The localization of the congruency effect seems to depend on the task and contrast used. For example, left hemisphere emphasis was found using more complex stimulation with six different syllables (Arnal et al., 2009) and left frontotemporal regions for symbol–speech sound comparisons (Xu et al., 2019).

The direction of the congruency effect was also in line with earlier studies using audio-visual stimuli showing more brain activity for the incongruent compared to the congruent stimuli (e.g., Arnal et al., 2009; Xu et al., 2019). The direction of the effect likely indicates the benefit of using two modalities to decode the speech signal reflected in less allocation of neuronal resources to the process when the two modalities match (e.g., Bernstein and Liebenthal, 2014). For the incongruent stimuli, the brain response likely includes an error detection signal for the mismatching auditory and visual input. Similar to Arnal et al. (2009), we compared responses to congruent and incongruent stimuli. In their study, they found significant differences in relatively late time-windows, which showed multiple steps for audio-visual processing (with differences at ∼250, ∼370, and ∼460 ms, with responses being larger for the congruent stimuli at the first time-point, and larger for the incongruent stimuli at the later time-points) localized to the auditory cortex and the STS.

The lack of congruency effects in the time-windows after 400 ms in this study could be due to the differences in the complexity of the experimental design used, the features of the stimulus material and the timing parameters between the auditory and visual features of the present study and earlier studies. For example, in Arnal et al. (2009) audio-visual combinations of five different syllables were used, which made the identification of congruency more difficult and possibly required further processing steps compared to the current study.

Furthermore, we found no early effects of congruency at N1 m response (75–25 ms following sound onset), which is in line with previous observations (Stekelenburg and Vroomen, 2007). Our results corroborate the assumption that early responses are predominantly sensitive to the stimulus material used for the comparisons. Differences found in the N1 and P2 time-windows were related to suppression effects of audio-visual stimuli compared to audio only stimuli, and not to the direct comparison of congruent and incongruent audio-visual stimuli (van Wassenhove et al., 2005; Stekelenburg and Vroomen, 2007).

The source localization result of the current study was in line with the sensor-level results in terms of the time-window. However, the clusters at the source level were observed only in the right hemisphere and in a widely spread area encompassing the superior temporal areas as well as the medial and ventral surfaces of the temporal lobe. The superior temporal cortex is roughly in line with that found in Arnal et al. (2009). The widely spread clustering at the source level could be due to methodological limitations. It is important to note that we used a template MRI, and this could have increased the localization error of the brain responses in the source-level analysis. Furthermore, the difference was found in a relatively late time-window and appears quite widespread in time, and the localization of ongoing activation can be more challenging than those of clear time-locked evoked responses. These might explain the differences in the locations of the clusters between the sensor and source level, although we assume they reflect the same effect.

We found no significant effects of familiarity when directly comparing the responses to stimuli that were part of the participants’ native language and to stimuli that were not part of their native language. The earlier studies have mostly examined this in auditory oddball experiments investigating deviance detection based on categorical perception of phonemes (e.g., Näätänen et al., 1997; Winkler et al., 1999). First, having equal probabilities of presentation for each stimulus type allows examination of the obligatory sensory responses without overlap from other processes. However, our null results comparing the responses to these stimuli in a passive cross-modal task suggest that the use of either an active comparison involving phonological representations or an identification task which would actively engage these representations is needed to lead to differences in brain activity for familiar and unfamiliar speech stimuli. Second, we examined evoked responses to audio-visual stimuli instead of induced brain activity. It is possible that the familiarity effects could produce brain activity that is not phase-locked to the stimuli. In this case the effect would not be observable in evoked responses. However, we did not have a hypothesis on the specific frequency band or time-window, where the difference in induced activity could be observed. Future studies could examine this in more detail.

The familiarity of speech in our study referred to whether participants perceiving the stimuli had prior knowledge of them, i.e., whether the syllables were present in their native phonology or not. Our stimuli (syllables) were produced by a native Chinese, non-finish speaker. This was required as native Finnish speakers would not be able to naturally produce all stimuli used in the experiment. Future studies could examine the effect of the speaker identity by using recordings of both native Chinese speaker and native Finnish speaker and how it might interact with the phonological familiarity of speech sounds.

## Conclusion

Our results show that in the case of audio-visual speech stimuli, congruency has an effect around 300 to 400 ms after the start of voicing. This effect was found in the temporal-parietal brain areas, partly replicating earlier findings. We found no significant differences between Chinese and Finnish speakers in their brain responses depending on the familiarity of the speech stimuli, that is, whether the syllables belonged to the native language or not. This suggests that the congruency effect is a result of a general detection of a mismatch between prediction based on lip movements and the auditory signal.

## Ethics Statement

This study was carried out in accordance with the recommendations of Ethics Committee of the University of Jyväskylä with written informed consent from all subjects. All subjects gave written informed consent in accordance with the Declaration of Helsinki. The protocol was approved by the Ethics Committee of the University of Jyväskylä.

## Author Contributions

OK, JH, and WX designed the study, performed the MEG experiments, and analyzed the data. All authors discussed the results and contributed to the final manuscript.

## Conflict of Interest Statement

The authors declare that the research was conducted in the absence of any commercial or financial relationships that could be construed as a potential conflict of interest.

## Supplementary Material

FIGURE S1 Grand average plots at sensor and source level for incongruent and congruent audio-visual stimuli for the two groups. **(a)** Grand averaged waveform for the combined planar gradient (vector sum of the paired orthogonal gradiometer channels) channels grouped (channels included indicated by circles) over the left and right temporal channels in the Chinese (above, *N*=12) and Finnish (below, *N*=12) groups. **(b)** Magnetic field topography and dynamic statistical parametric maps (dSPM) source activation of the grand average evoked responses in the five time-windows investigated in the study (75–125, 150–200, 200–300, 300–400, and 400–600 ms) for the two conditions.

FIGURE S2 Grand average plots at sensor and source level for unfamiliar and familiar congruent audio-visual stimuli for the two groups. **(a)** Grand averaged waveform for the combined planar gradient (vector sum of the paired orthogonal gradiometer channels) channels grouped (channels included indicated by circles) over the left and right temporal channels in the Chinese (above, *N*=12) and Finnish (below, *N*=12) groups. **(b)** Magnetic field topography and dynamic statistical parametric maps (dSPM) source activation of the grand average evoked responses in the five time-windows investigated in the study (75–125, 150–200, 200–300, 300–400, and 400–600 ms) for the two conditions.
