# Supplementary figures and images for: Top-Down Predictions of Familiarity and Congruency in Audio-Visual Speech Perception at Neural Level
Source: Front Hum Neurosci. 2019 Jul 12;13:243. doi: 10.3389/fnhum.2019.00243 (PMC6639789; doi:10.3389/fnhum.2019.00243)

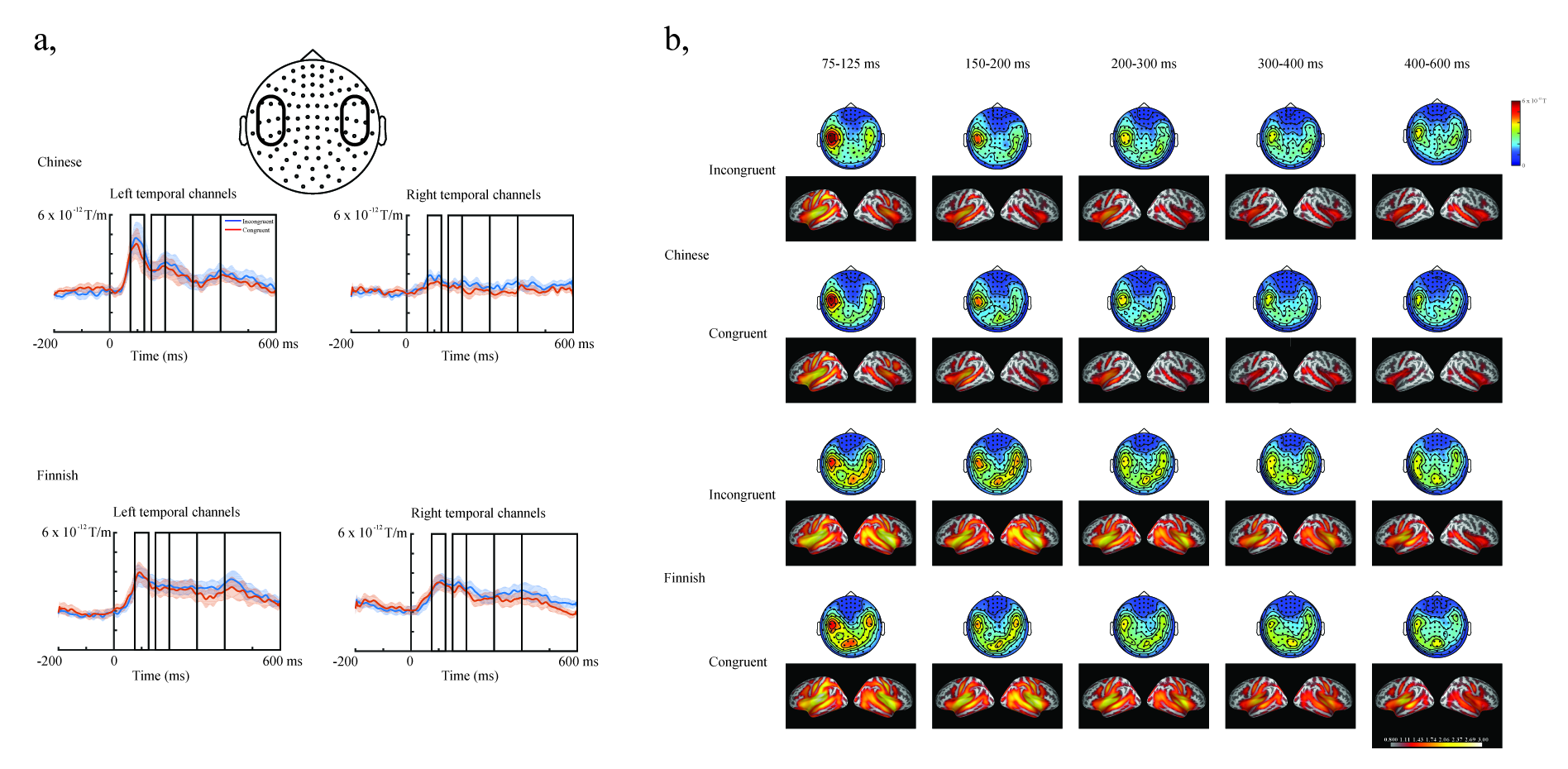

Supplement: FIGURE S1 — Grand average plots at sensor and source level for incongruent and congruent audio-visual stimuli for the two groups. (a) Grand averaged waveform for the combined planar gradient (vector sum of the paired orthogonal gradiometer channels) channels grouped (channels included indicated by circles) over the left and right temporal channels in the Chinese (above, N=12) and Finnish (below, N=12) groups. (b) Magnetic field topography and dynamic statistical parametric maps (dSPM) source activation of the grand average evoked responses in the five time-windows investigated in the study (75–125, 150–200, 200–300, 300–400, and 400–600 ms) for the two conditions. [file Image_1.TIF]

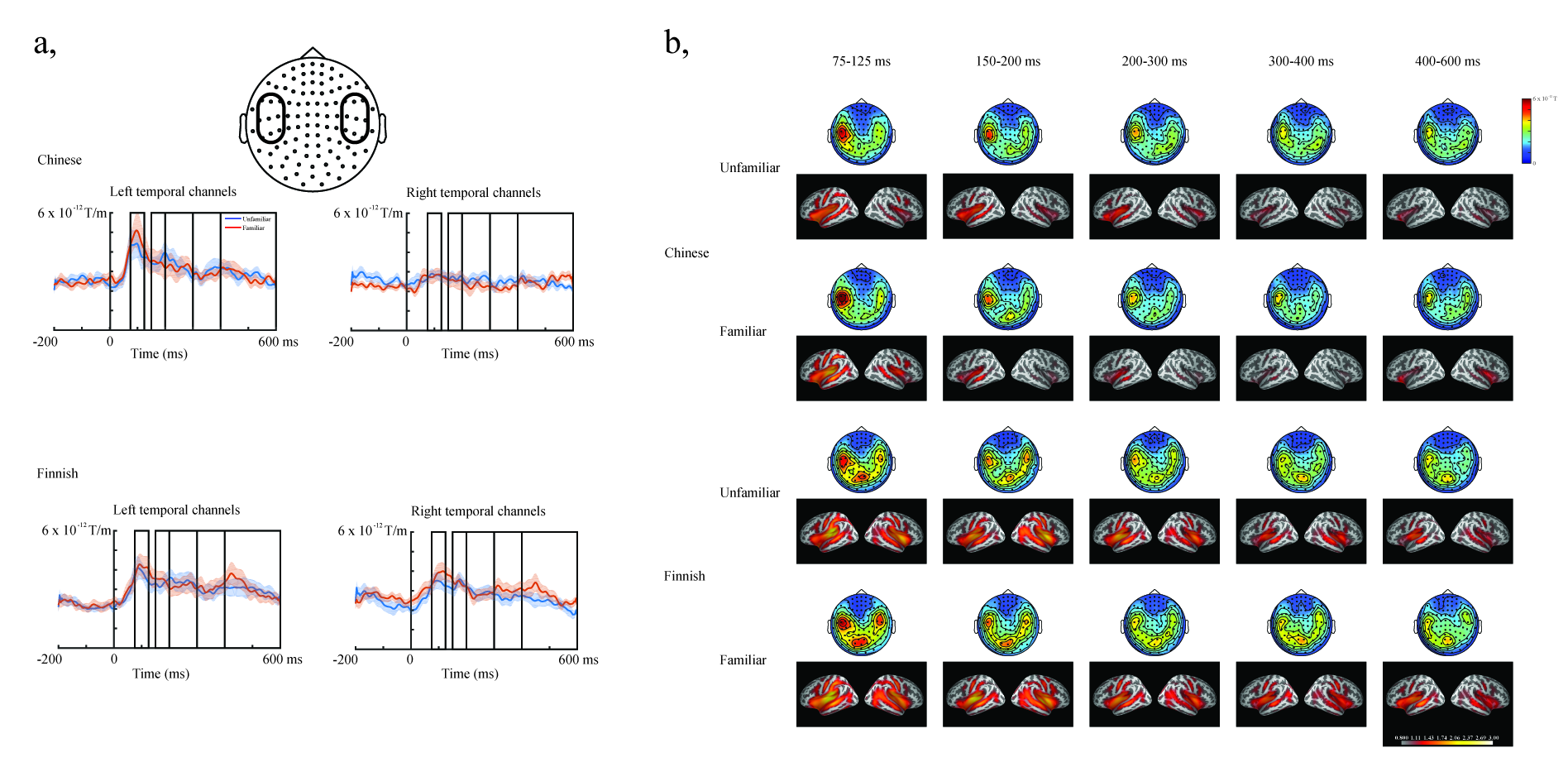

Supplement: FIGURE S2 — Grand average plots at sensor and source level for unfamiliar and familiar congruent audio-visual stimuli for the two groups. (a) Grand averaged waveform for the combined planar gradient (vector sum of the paired orthogonal gradiometer channels) channels grouped (channels included indicated by circles) over the left and right temporal channels in the Chinese (above, N=12) and Finnish (below, N=12) groups. (b) Magnetic field topography and dynamic statistical parametric maps (dSPM) source activation of the grand average evoked responses in the five time-windows investigated in the study (75–125, 150–200, 200–300, 300–400, and 400–600 ms) for the two conditions. [file Image_2.TIF]
