# Supplementary material for: Top-Down Predictions of Familiarity and Congruency in Audio-Visual Speech Perception at Neural Level
Source: Front Hum Neurosci. 2019 Jul 12;13:243. doi: 10.3389/fnhum.2019.00243 (PMC6639789; doi:10.3389/fnhum.2019.00243)
Supplement: Supplementary file 3 [file Data_Sheet_1.pdf]

Supplementary material – Permutation tests results

Sensor level:

Congruence:

No significant effects were found in the first, second, third and fifth time-windows.

For the Stimulus type x Native language interaction, 14 clusters were found in the first time-window and the lowest *p*-value was 0.599. In the second time-window, 3 clusters were found and the lowest p-value was 0.744. In the third time-window, 15 clusters were found and the lowest p-value was 1. In the fourth time-window, 16 clusters were found and the lowest p-value was 0.693. In the fifth time-window, 42 clusters were found and the lowest p-value was 0.965.

For Stimulus type (Congruency) main effect, 12 clusters were found in the first-time window and the lowest p-value was 0.117. In the second time-window, 15 clusters were found and the lowest p-value was 0.802. In the third time-window, 21 clusters were found and the lowest p-value was 0.083. In the fourth time-window 12 clusters were found, two of which were significant and the p-values and topographies have been reported in the manuscript. In the fifth time-window, 37 clusters were found and the lowest p-value was 0.069.

Familiarity:

No significant statistical effects were found in the five time-windows examined using the cluster permutation tests.

For the Stimulus type x Native language interaction, 9 clusters were found in the first time-window and the lowest p-value was 0.402. In the second time-window, 12 clusters were found and the lowest p-value was 0.275. In the third time-window, 20 clusters were found and the lowest p-value was 0.426. In the fourth time-window, 27 clusters were found and the lowest p-value was 0.171. In the fifth time-window, 34 clusters were found and the lowest p-value was 0.095.

For Stimulus type (Familiarity) main effect, 9 clusters were found in the first-time window, and the lowest p-value was 0.330. In the second time-window, 11 clusters were found and the lowest p-value was 0.990. In the third time-window, 12 clusters were found and the lowest p-value was 0.371. In the fourth time-window, 17 clusters were found and the lowest p-value was 0.220.In the fifth time-window, 36 clusters were found and the lowest p-value was 0.107.

Source level:

Congruence:

No significant effects were found in the first, second, third and fifth time-windows.

For the Stimulus type x Native language interaction, 87 clusters were found in the first time-window and the lowest p-value was 0.689. In the second time-window, 81 clusters were found and the lowest p-value was 0.763. In the third time-window, 153 clusters were found and the lowest p-value was 0.787. In the fourth time-window, 147 clusters were found and the lowest p-value was 0.926. In the fifth time-window, 265 clusters were found and the lowest p-value was 0.705.

For Stimulus type (Congruency) main effect, 76 clusters were found in the first-time window and the lowest p-value was 0.870. In the second time-window, 67 clusters were found and the lowest p-value was 0.991. In the third time-window, 133 clusters were found and the lowest p-value was 0.309. In the fourth time-window 110 clusters were found, one of which was significant and the p-value and topography have been reported in the manuscript. In the fifth time-window, 259 clusters were found and the lowest p-value was 0.281.

Familiarity:

No significant statistical effects were found in the five time-windows examined using the cluster permutation tests.

For the Stimulus type x Native language interaction, 87 clusters were found in the first time-window and the lowest p-value was 0.684. In the second time-window, 81 clusters were found and the lowest p-value was 0.766. In the third time-window, 153 clusters were found and the lowest p-value was 0.784. In the fourth time-window, 147 clusters were found and the lowest p-value was 0.915. In the fifth time-window, 265 clusters were found and the lowest p-value was 0.710.

For Stimulus type (Familiarity) main effect, 50 clusters were found in the first-time window and the lowest p-value was 0.194. In the second time-window, 64 clusters were found and the lowest p-value was 0.590. In the third time-window, 138 clusters were found and the lowest p-value was 0.646. In the fourth time-window, 137 clusters were found and the lowest p-value was 0.775. In the fifth time-window, 242 clusters were found and the lowest p-value was 0.258.
